# (*E*,*E*)-3,3′-Dimethyl-1,1′-diphenyl-4,4′-{(ethane-1,2-diyldiimino)­bis­[(2-fur­yl)methyl­idyne]}di-1*H*-pyrazol-5(4*H*)-one

**DOI:** 10.1107/S160053681001980X

**Published:** 2010-06-05

**Authors:** Hai-Wen Wang

**Affiliations:** a East China University of Science and Technology, Collage of Chemistry and Molecular Engineering, Mei Long Road 130, Shanghai 200237, People’s Republic of China

## Abstract

The complete molecule of the title compound of the title compound, C_32_H_28_N_6_O_4_, is generated by crystallographic inversion symmetry.  The dihedral angles between the pyrazalone ring and the pendant phenyl and furan rings are 15.65 (8) and 65.06 (8)°, respectively.  In the crystal, the molecules are linked by N—H⋯O, C—H⋯O and weak C—H⋯π interactions.

## Related literature

For general background to pyrazolo­nes, see: Casas *et al.* (2007 ▶); Jensen (1959 ▶); Li *et al.* (2000 ▶); Zhang *et al.* (2007 ▶, 2008 ▶).
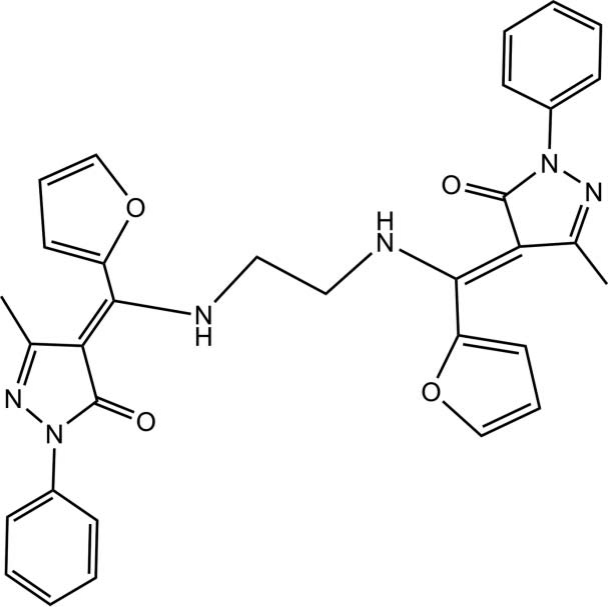


## Experimental

### 

#### Crystal data

C_32_H_28_N_6_O_4_
*M* *_r_* = 560.60
Monoclinic, 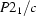

*a* = 10.7438 (6) Å
*b* = 7.6999 (4) Å
*c* = 16.8273 (9) Å
β = 93.937 (1)°
*V* = 1388.77 (13) Å^3^
*Z* = 2
Mo *K*α radiation
μ = 0.09 mm^−1^
*T* = 295 K
0.22 × 0.20 × 0.20 mm

#### Data collection

Bruker APEXII CCD area-detector diffractometer
8081 measured reflections
3145 independent reflections
1934 reflections with *I* > 2σ(*I*)
*R*_int_ = 0.026

#### Refinement

*R*[*F* ^2^ > 2σ(*F* ^2^)] = 0.046
*wR*(*F* ^2^) = 0.133
*S* = 1.02
3145 reflections
195 parameters
H atoms treated by a mixture of independent and constrained refinement
Δρ_max_ = 0.16 e Å^−3^
Δρ_min_ = −0.21 e Å^−3^

### 

Data collection: *APEX2* (Bruker, 2005 ▶); cell refinement: *SAINT* (Bruker, 2005 ▶); data reduction: *SAINT*; program(s) used to solve structure: *SHELXS97* (Sheldrick, 2008 ▶); program(s) used to refine structure: *SHELXL97* (Sheldrick, 2008 ▶); molecular graphics: *SHELXTL* (Sheldrick, 2008 ▶); software used to prepare material for publication: *SHELXL97*.

## Supplementary Material

Crystal structure: contains datablocks I, global. DOI: 10.1107/S160053681001980X/zq2042sup1.cif

Structure factors: contains datablocks I. DOI: 10.1107/S160053681001980X/zq2042Isup2.hkl

Additional supplementary materials:  crystallographic information; 3D view; checkCIF report

## Figures and Tables

**Table 1:** Hydrogen-bond geometry (Å, °) *Cg*3 is the centroid of the C1–C6 ring.

*D*—H⋯*A*	*D*—H	H⋯*A*	*D*⋯*A*	*D*—H⋯*A*
N3—H3*A*⋯O1	0.92 (2)	1.91 (2)	2.693 (2)	142.0 (17)
C16—H16*A*⋯*Cg*3^i^	0.97	2.70	3.575 (2)	151
C3—H3⋯O1^ii^	0.93	2.54	3.389 (2)	152

Symmetry codes: (i) 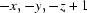; (ii) 
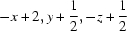
.

**Fig. 1.**
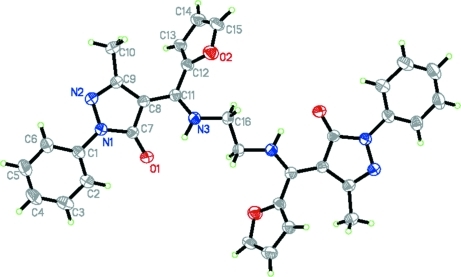
The molecular structure of (I) (thermal ellipsoids are shown at the 30% probability level). Symmetry code: (i) -x, y+1/2, -z+1/2.

## supplementary crystallographic information

### Comment

Pyrazolones form a very important class of heterocycles due to their properties and applications (Casas *et al.*, 2007). Schiffbases derived from 1-phenyl-3-methyl-4-(2-furoyl)-5-pyrazolone (PMFP) have found extensive application in coordination chemistry and in antibacterial activation (Zhang *et al.*, 2007, 2008; Li *et al.*, 2000). In order to expand this field, a novel bis-schiff base has been synthesized and its crystal structure is reported herein for the first time.

The molecular structure of the title compound (I) is shown in Fig.1. The molecule adopts a staggered conformation about the C9—C9^i ^bond (symmetry code: (i) -*x*, *y* + 1/2, -*z* + 1/2) due to the centrosymmetry. Atoms O1, C7, C8 and C11 of the PMFP moiety and N3 of the ethylenediamine group are almost coplanar, the largest deviation being 0.063 (11) Å for atom C11. The phenyl and furan rings are slightly twisted with respect to the central pyrazolone ring making dihedral angles of 15.65 (8)° and 65.06 (8)°, respectively, indicating a high degree of conjugation and electron delocalization.

The cohesion of the crystal is assured by a strong intramolecular hydrogen bond N3—H3A···O1 (Table 1). Three weak hydrogen bonds C2—H2···O1, C3—H3···O1 and C16–16 A···*Cg*(3) (*Cg*(3) denotes the C1—C6 ring centroid) also contribute to the stabilization of the crystal structure.

### Experimental

All reagents were obtained from commercial sources and used without further purification. PMFP was synthesized according to the method proposed by Jensen (Jensen, 1959). Ethylenediamine1.0 mmol (0.067 ml) was added dropwise to a stirred solution of PMFP 2.0 mmol (0.5365 g) in anhydrous ethanol (25 ml) at ambient temperature.After refluxing for 6 h, the solvent was removed and a pure yellowish product was obtained upon recrystallization from EtOH/n-heptane (V/V = 1) in 78% yield. mp: 181–182°C.

### Refinement

The H atom bonded to N3 was located in a difference map and refined freely. Other H atoms were placed in calculated positions, with C—H = 0.93 for phenyl, 0.96 for methyl and 0.97 Å for methylene H atoms, and refined as riding, with *U*_iso_(H) = 1.2*U*_eq_ (C) for phenyl and methylene H, and 1.5_eq_U(C) for methyl H atoms.

### Crystal data

**Table:**

C_32_H_28_N_6_O_4_	*F*(000) = 588
*M**_r_* = 560.60	*D*_x_ = 1.341 Mg m^−^^3^
Monoclinic, *P*2_1_/*c*	Mo *K*α radiation, λ = 0.71073 Å
Hall symbol: -P 2ybc	Cell parameters from 1740 reflections
*a* = 10.7438 (6) Å	θ = 2.4–22.9°
*b* = 7.6999 (4) Å	µ = 0.09 mm^−^^1^
*c* = 16.8273 (9) Å	*T* = 295 K
β = 93.937 (1)°	Block, yellow
*V* = 1388.77 (13) Å^3^	0.22 × 0.20 × 0.20 mm
*Z* = 2	

### Data collection

**Table:**

Bruker APEXII CCD area-detector diffractometer	1934 reflections with *I* > 2σ(*I*)
Radiation source: fine-focus sealed tube	*R*_int_ = 0.026
graphite	θ_max_ = 27.5°, θ_min_ = 1.9°
phi and ω scans	*h* = −12→13
8081 measured reflections	*k* = −10→6
3145 independent reflections	*l* = −21→21

### Refinement

**Table:**

Refinement on *F*^2^	Primary atom site location: structure-invariant direct methods
Least-squares matrix: full	Secondary atom site location: difference Fourier map
*R*[*F*^2^ > 2σ(*F*^2^)] = 0.046	Hydrogen site location: inferred from neighbouring sites
*wR*(*F*^2^) = 0.133	H atoms treated by a mixture of independent and constrained refinement
*S* = 1.01	*w* = 1/[σ^2^(*F*_o_^2^) + (0.0585*P*)^2^ + 0.2282*P*] where *P* = (*F*_o_^2^ + 2*F*_c_^2^)/3
3145 reflections	(Δ/σ)_max_ < 0.001
195 parameters	Δρ_max_ = 0.16 e Å^−^^3^
0 restraints	Δρ_min_ = −0.21 e Å^−^^3^

### Special details

**Table:**

Geometry. All e.s.d.'s (except the e.s.d. in the dihedral angle between two l.s. planes) are estimated using the full covariance matrix. The cell e.s.d.'s are taken into account individually in the estimation of e.s.d.'s in distances, angles and torsion angles; correlations between e.s.d.'s in cell parameters are only used when they are defined by crystal symmetry. An approximate (isotropic) treatment of cell e.s.d.'s is used for estimating e.s.d.'s involving l.s. planes.
Refinement. Refinement of *F*^2^ against ALL reflections. The weighted *R*-factor *wR* and goodness of fit *S* are based on *F*^2^, conventional *R*-factors *R* are based on *F*, with *F* set to zero for negative *F*^2^. The threshold expression of *F*^2^ > σ(*F*^2^) is used only for calculating *R*-factors(gt) *etc*. and is not relevant to the choice of reflections for refinement. *R*-factors based on *F*^2^ are statistically about twice as large as those based on *F*, and *R*- factors based on ALL data will be even larger.

### Fractional atomic coordinates and isotropic or equivalent isotropic displacement parameters (Å^2^)

**Table:**

	*x*	*y*	*z*	*U*_iso_*/*U*_eq_	
C1	0.79340 (17)	1.2879 (2)	0.11911 (11)	0.0503 (4)	
C2	0.86997 (19)	1.2531 (3)	0.18661 (11)	0.0628 (5)	
H2	0.9052	1.1435	0.1942	0.075*	
C3	0.8939 (2)	1.3819 (4)	0.24275 (14)	0.0784 (7)	
H3	0.9448	1.3584	0.2884	0.094*	
C4	0.8430 (2)	1.5442 (4)	0.23149 (16)	0.0832 (8)	
H4	0.8600	1.6308	0.2692	0.100*	
C5	0.7671 (2)	1.5788 (3)	0.16450 (15)	0.0755 (7)	
H5	0.7329	1.6890	0.1570	0.091*	
C6	0.74101 (18)	1.4516 (3)	0.10811 (13)	0.0598 (5)	
H6	0.6887	1.4753	0.0631	0.072*	
C7	0.83098 (17)	1.0057 (2)	0.04804 (11)	0.0494 (4)	
C8	0.76317 (16)	0.9266 (2)	−0.01917 (10)	0.0477 (4)	
C9	0.66731 (17)	1.0489 (3)	−0.04361 (11)	0.0529 (5)	
C10	0.5737 (2)	1.0478 (3)	−0.11395 (13)	0.0757 (7)	
H10A	0.5098	0.9640	−0.1054	0.114*	
H10B	0.6145	1.0178	−0.1610	0.114*	
H10C	0.5369	1.1610	−0.1204	0.114*	
C11	0.79841 (15)	0.7652 (2)	−0.04839 (10)	0.0453 (4)	
C12	0.71716 (18)	0.6668 (2)	−0.10479 (10)	0.0510 (5)	
C13	0.59548 (17)	0.6410 (3)	−0.10780 (11)	0.0566 (5)	
H13	0.5408	0.6837	−0.0720	0.068*	
C14	0.5636 (2)	0.5375 (3)	−0.17468 (13)	0.0728 (6)	
H14	0.4841	0.4988	−0.1915	0.087*	
C15	0.6675 (2)	0.5054 (3)	−0.20900 (13)	0.0743 (6)	
H15	0.6733	0.4402	−0.2551	0.089*	
C16	0.95252 (16)	0.5224 (2)	−0.03384 (11)	0.0498 (5)	
H16A	0.9908	0.5153	−0.0843	0.060*	
H16B	0.8836	0.4408	−0.0350	0.060*	
H3A	0.9502 (18)	0.768 (3)	0.0148 (12)	0.072 (6)*	
N1	0.76746 (14)	1.1578 (2)	0.06110 (8)	0.0525 (4)	
N2	0.67014 (15)	1.1852 (2)	0.00285 (9)	0.0586 (4)	
N3	0.90719 (14)	0.6974 (2)	−0.02175 (10)	0.0523 (4)	
O1	0.92691 (12)	0.95452 (17)	0.08686 (8)	0.0615 (4)	
O2	0.76644 (13)	0.5832 (2)	−0.16638 (8)	0.0703 (4)	

### Atomic displacement parameters (Å^2^)

**Table:**

	*U*^11^	*U*^22^	*U*^33^	*U*^12^	*U*^13^	*U*^23^
C1	0.0526 (10)	0.0503 (11)	0.0489 (10)	−0.0084 (9)	0.0096 (8)	−0.0029 (9)
C2	0.0687 (13)	0.0644 (13)	0.0550 (11)	−0.0098 (11)	0.0030 (10)	−0.0045 (10)
C3	0.0795 (15)	0.0934 (19)	0.0622 (14)	−0.0174 (14)	0.0041 (11)	−0.0200 (13)
C4	0.0753 (15)	0.0879 (19)	0.0887 (18)	−0.0172 (14)	0.0219 (14)	−0.0418 (15)
C5	0.0656 (13)	0.0618 (14)	0.1014 (18)	−0.0080 (11)	0.0215 (13)	−0.0244 (13)
C6	0.0572 (11)	0.0562 (12)	0.0673 (12)	−0.0035 (10)	0.0143 (10)	−0.0061 (10)
C7	0.0528 (10)	0.0465 (10)	0.0481 (10)	0.0005 (9)	−0.0017 (8)	0.0039 (8)
C8	0.0505 (10)	0.0465 (10)	0.0452 (9)	0.0000 (8)	−0.0029 (8)	0.0006 (8)
C9	0.0550 (11)	0.0534 (11)	0.0490 (10)	0.0044 (9)	−0.0051 (8)	0.0015 (9)
C10	0.0822 (15)	0.0761 (16)	0.0647 (13)	0.0176 (13)	−0.0249 (11)	−0.0040 (12)
C11	0.0472 (10)	0.0460 (10)	0.0426 (9)	−0.0017 (8)	0.0022 (8)	0.0051 (8)
C12	0.0622 (12)	0.0480 (10)	0.0425 (9)	−0.0011 (9)	0.0002 (8)	−0.0025 (8)
C13	0.0475 (10)	0.0667 (13)	0.0559 (11)	0.0026 (9)	0.0049 (8)	−0.0122 (10)
C14	0.0565 (12)	0.0862 (17)	0.0741 (14)	−0.0062 (12)	−0.0086 (11)	−0.0161 (12)
C15	0.0718 (14)	0.0852 (16)	0.0641 (13)	−0.0018 (13)	−0.0076 (11)	−0.0288 (12)
C16	0.0482 (10)	0.0454 (10)	0.0561 (11)	−0.0007 (8)	0.0052 (8)	−0.0022 (8)
N1	0.0594 (9)	0.0474 (9)	0.0494 (9)	0.0036 (8)	−0.0063 (7)	−0.0026 (7)
N2	0.0625 (10)	0.0552 (10)	0.0564 (9)	0.0090 (8)	−0.0087 (8)	−0.0015 (8)
N3	0.0497 (9)	0.0426 (9)	0.0636 (10)	−0.0014 (7)	−0.0020 (8)	−0.0042 (8)
O1	0.0619 (8)	0.0561 (8)	0.0635 (8)	0.0046 (7)	−0.0177 (7)	−0.0037 (7)
O2	0.0630 (9)	0.0847 (11)	0.0632 (9)	0.0002 (8)	0.0047 (7)	−0.0147 (8)

### Geometric parameters (Å, °)

**Table:**

C1—C2	1.382 (3)	C10—H10A	0.9600
C1—C6	1.387 (3)	C10—H10B	0.9600
C1—N1	1.413 (2)	C10—H10C	0.9600
C2—C3	1.382 (3)	C11—N3	1.330 (2)
C2—H2	0.9300	C11—C12	1.457 (2)
C3—C4	1.372 (4)	C12—C13	1.320 (2)
C3—H3	0.9300	C12—O2	1.358 (2)
C4—C5	1.371 (4)	C13—C14	1.402 (3)
C4—H4	0.9300	C13—H13	0.9300
C5—C6	1.379 (3)	C14—C15	1.315 (3)
C5—H5	0.9300	C14—H14	0.9300
C6—H6	0.9300	C15—O2	1.378 (2)
C7—O1	1.246 (2)	C15—H15	0.9300
C7—N1	1.380 (2)	C16—N3	1.452 (2)
C7—C8	1.438 (2)	C16—C16^i^	1.516 (3)
C8—C11	1.398 (3)	C16—H16A	0.9700
C8—C9	1.435 (2)	C16—H16B	0.9700
C9—N2	1.307 (2)	N1—N2	1.399 (2)
C9—C10	1.500 (2)	N3—H3A	0.92 (2)
			
C2—C1—C6	119.95 (18)	H10A—C10—H10C	109.5
C2—C1—N1	120.52 (18)	H10B—C10—H10C	109.5
C6—C1—N1	119.54 (17)	N3—C11—C8	118.92 (16)
C3—C2—C1	119.7 (2)	N3—C11—C12	119.25 (17)
C3—C2—H2	120.2	C8—C11—C12	121.79 (16)
C1—C2—H2	120.2	C13—C12—O2	109.65 (16)
C4—C3—C2	120.4 (2)	C13—C12—C11	130.61 (17)
C4—C3—H3	119.8	O2—C12—C11	119.74 (16)
C2—C3—H3	119.8	C12—C13—C14	107.65 (18)
C5—C4—C3	119.9 (2)	C12—C13—H13	126.2
C5—C4—H4	120.0	C14—C13—H13	126.2
C3—C4—H4	120.0	C15—C14—C13	106.94 (18)
C4—C5—C6	120.6 (2)	C15—C14—H14	126.5
C4—C5—H5	119.7	C13—C14—H14	126.5
C6—C5—H5	119.7	C14—C15—O2	109.74 (18)
C5—C6—C1	119.5 (2)	C14—C15—H15	125.1
C5—C6—H6	120.3	O2—C15—H15	125.1
C1—C6—H6	120.3	N3—C16—C16^i^	108.70 (18)
O1—C7—N1	125.83 (17)	N3—C16—H16A	109.9
O1—C7—C8	129.37 (17)	C16^i^—C16—H16A	109.9
N1—C7—C8	104.79 (15)	N3—C16—H16B	109.9
C11—C8—C9	133.55 (16)	C16^i^—C16—H16B	109.9
C11—C8—C7	121.23 (16)	H16A—C16—H16B	108.3
C9—C8—C7	105.20 (16)	C7—N1—N2	111.73 (14)
N2—C9—C8	111.62 (16)	C7—N1—C1	129.44 (15)
N2—C9—C10	117.60 (17)	N2—N1—C1	118.64 (15)
C8—C9—C10	130.68 (18)	C9—N2—N1	106.47 (15)
C9—C10—H10A	109.5	C11—N3—C16	127.80 (16)
C9—C10—H10B	109.5	C11—N3—H3A	112.4 (12)
H10A—C10—H10B	109.5	C16—N3—H3A	119.0 (13)
C9—C10—H10C	109.5	C12—O2—C15	106.01 (15)
			
C6—C1—C2—C3	−0.1 (3)	C8—C11—C12—O2	139.07 (18)
N1—C1—C2—C3	−179.80 (18)	O2—C12—C13—C14	−0.6 (2)
C1—C2—C3—C4	−0.6 (3)	C11—C12—C13—C14	179.5 (2)
C2—C3—C4—C5	0.6 (4)	C12—C13—C14—C15	0.0 (3)
C3—C4—C5—C6	0.1 (3)	C13—C14—C15—O2	0.6 (3)
C4—C5—C6—C1	−0.7 (3)	O1—C7—N1—N2	174.61 (17)
C2—C1—C6—C5	0.7 (3)	C8—C7—N1—N2	−4.5 (2)
N1—C1—C6—C5	−179.56 (17)	O1—C7—N1—C1	−0.1 (3)
O1—C7—C8—C11	3.0 (3)	C8—C7—N1—C1	−179.26 (17)
N1—C7—C8—C11	−177.92 (16)	C2—C1—N1—C7	−19.0 (3)
O1—C7—C8—C9	−175.32 (19)	C6—C1—N1—C7	161.30 (18)
N1—C7—C8—C9	3.75 (19)	C2—C1—N1—N2	166.60 (16)
C11—C8—C9—N2	−179.9 (2)	C6—C1—N1—N2	−13.1 (2)
C7—C8—C9—N2	−1.9 (2)	C8—C9—N2—N1	−0.8 (2)
C11—C8—C9—C10	−3.7 (4)	C10—C9—N2—N1	−177.56 (17)
C7—C8—C9—C10	174.3 (2)	C7—N1—N2—C9	3.4 (2)
C9—C8—C11—N3	167.09 (19)	C1—N1—N2—C9	178.82 (16)
C7—C8—C11—N3	−10.7 (3)	C8—C11—N3—C16	169.17 (17)
C9—C8—C11—C12	−15.3 (3)	C12—C11—N3—C16	−8.5 (3)
C7—C8—C11—C12	166.92 (16)	C16^i^—C16—N3—C11	−154.74 (19)
N3—C11—C12—C13	136.6 (2)	C13—C12—O2—C15	0.9 (2)
C8—C11—C12—C13	−41.0 (3)	C11—C12—O2—C15	−179.14 (18)
N3—C11—C12—O2	−43.3 (2)	C14—C15—O2—C12	−0.9 (3)

Symmetry codes: (i) −*x*+2, −*y*+1, −*z*.

### Hydrogen-bond geometry (Å, °)

**Table:**

Cg3 is the centroid of the C1–c6 ring.

**Table:**

*D*—H···*A*	*D*—H	H···*A*	*D*···*A*	*D*—H···*A*
N3—H3A···O1	0.92 (2)	1.91 (2)	2.693 (2)	142.0 (17)
C16—H16A···Cg3^ii^	0.97	2.70	3.575 (2)	151
C3—H3···O1^iii^	0.93	2.54	3.389 (2)	152

Symmetry codes:  (ii) −*x*, −*y*, −*z*+1; (iii) −*x*+2, *y*+1/2, −*z*+1/2.
